# Rapid shallow megathrust afterslip from the 2021 M8.2 Chignik, Alaska earthquake revealed by seafloor geodesy

**DOI:** 10.1126/sciadv.adf9299

**Published:** 2023-04-26

**Authors:** Benjamin A. Brooks, Dara Goldberg, John DeSanto, Todd L. Ericksen, Spahr C. Webb, Scott L. Nooner, C. David Chadwell, James Foster, Sarah Minson, Robert Witter, Peter Haeussler, Jeffrey Freymueller, William Barnhart, Johanna Nevitt

**Affiliations:** ^1^ Earthquake Science Center, U.S. Geological Survey, Moffett Field, CA, USA.; ^2^ National Earthquake Information Center, Geological Hazards Science Center, U.S. Geological Survey, Golden, CO, USA.; ^3^ University of Washington, Seattle, WA, USA.; ^4^ Lamont-Doherty Earth Observatory, Palisades, NY, USA.; ^5^ University of North Carolina Wilmington, Wilmington, NC, USA.; ^6^ Scripps Institution of Oceanography, La Jolla, CA, USA.; ^7^ Stuttgart University, Stuttgart, Germany.; ^8^ Alaska Science Center, U.S. Geological Survey, Anchorage, AK, USA.; ^9^ Michigan State University, East Lansing, MI, USA.

## Abstract

The shallower portions of subduction zone megathrust faults host Earth’s most hazardous tsunamigenic earthquakes, yet understanding how and when they slip remains elusive because of challenges making seafloor observations. We performed Global Navigation Satellite System Acoustic seafloor geodetic surveys before and ~2.5 months after the 29 July 2021 *M*_w_ (moment magnitude) 8.2 Chignik, Alaska, earthquake and determine ~1.4 meters cumulative co- and post-seismic horizontal displacement ~60 kilometers from the megathrust front. Only for the 2011 *M*_w_ 9 Tohoku event have closer subduction zone earthquake displacements been observed. We estimate ~2 to 3 meters of megathrust afterslip shallower than 20 kilometers, a portion of the megathrust on which both inter- and co-seismic slip likely had occurred previously. Our analysis demonstrates that by 2.5 months, shallower and deeper moment had effectively equilibrated on the megathrust, suggesting that its tsunamigenic potential remains no more elevated than before the earthquake.

## Teaser

Seafloor geodesy reveals rapid up-dip afterslip following the *M*_w_ 8.2 Chignik subduction zone earthquake.

## INTRODUCTION

**Fig. 1.**
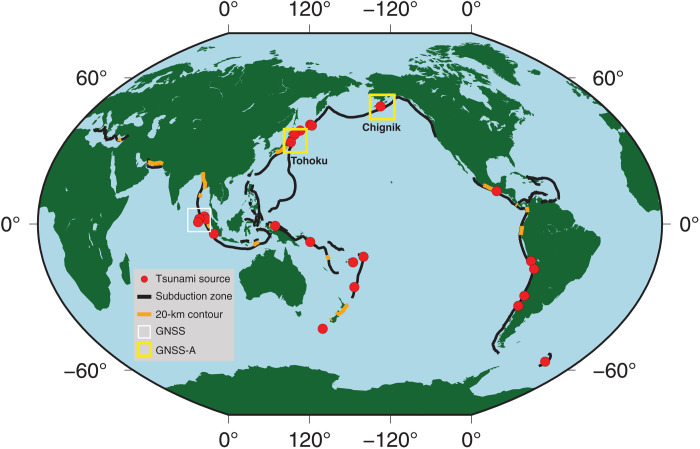
Megathrust observing capacity. Global distribution of tsunamigenic sources since 1990 (red circles) (*6*), subduction zones (black lines), and locations where there is land that could host geodetic instrumentation over the subduction zone 20-km-depth contour (orange lines) (*19*). Boxes indicate where geodetic measurements above, or shallower than, the 20-km subduction zone contour have been made for tsunamigenic earthquakes. White box, GNSS data only (*7*, *9*, *14*); yellow boxes, GNSS and GNSS-Acoustic (GNSS-A) data for Tohoku (*14*) and this study.

Subduction zone earthquakes and tsunami are some of Earth’s most impactful and societally relevant natural hazards (*1*). Despite this heightened profile, however, understanding of the governing physical processes and our ability to assess the space-time evolution of subduction zones’ coupled earthquake and tsunami hazard remains limited due to difficulty making observations in these challenging regions (Fig. 1) (*2*). In particular, the shallower part of subduction zones where the underlying megathrust fault plane extends toward the seafloor is very poorly observed and understood. Perhaps, the most pressing issue connecting process and hazard is determining the frictional properties of the megathrust, and under what circumstances subduction zone earthquake fault slip may propagate along the megathrust and any of its subsidiary splay faults from nucleation depths (typically greater than 10 to 20 km) to the surface where the seafloor can be most dynamically deformed and tsunamigenic (*3*, *4*). Since 1990, roughly, the time when land-based Global Navigation Satellite Systems (GNSS) geodetic networks began to yield centimeter-scale or better surface displacement capability (*5*), there have been 23 subduction zone earthquakes large enough to create measurable tsunami (*6*), yet for only three events do we have even a few geodetic measurements directly above megathrust depths of 20 km or shallower (Fig. 1) (*7*–*9*). Even if they are eventually monumented with GNSS networks, only a very small portion of subduction zones have land above or outboard of the 20-km megathrust contour (Fig. 1).

It often has been assumed that earthquake ruptures were inhibited by shallow megathrust sections tending to exhibit stable, “velocity strengthening (VS),” frictional character (*10*). Consistent with this view, slip models of the M9 Sumatra-Andaman earthquake and its >M8 aftershocks exhibit complementary, nonoverlapping, deep co-seismic slip and shallow afterslip on the megathrust (*7*, *11*, *12*). More recently, however, geodetic inference of precursory stable sliding and co-seismic slip during the 2011 M9 Tohoku earthquake indicates that the same intermediate depth portion of a megathrust can host rapid co-seismic slip indicative of velocity weakening (VW) frictional character, as well as slower, stable, or conditionally stable slip (*13*–*15*). This recognition of bimodal types of slip behavior for the same portion of the megathrust stimulated theoretical developments that provide potential physical explanations for a VS portion of a patch to also exhibit VW behavior (*16*–*18*).

Further complicating matters, especially for hazards assessment, evaluating megathrust slip potency has relied on estimating comparative slip deficits inferred from land-based geodetic networks, which are almost universally located above the deeper portions of megathrusts (*2*). As part of its natural hazards program, the U.S. Geological Survey (USGS) responds to and provides rapid assessments of the impacts and evolving hazards of global earthquakes (*19*–*21*). This includes automatically producing slip models (“finite fault models”) from teleseismic waveforms (*22*, *23*); recent efforts are focused on refining the models by including geodetic surface displacement data (*24*). For subduction zone earthquakes, finite fault models (FFMs) are used to rapidly assess which portions of the megathrust have slipped and which portions are stressed and/or retain potentially seismo- and tsunamigenic slip deficits. Post-seismic afterslip (*25*, *26*) can act to reduce slip deficit and tsunami potential for shallow megathrusts. On land, earthquake response prioritizes deploying seismic and geodetic instrumentation as close as possible to the responsible fault planes to monitor afterslip (*27*, *28*). Recently, the growth of seafloor geodetic observational capability permits similar observations to be made for the submarine portions of subduction zone megathrusts (*29*–*32*), albeit with longer response times due to the logistical complexities of ocean-going operations. To date, seafloor geodetic observations have been made in association with only one earthquake, the M9 2011 Tohoku event (*13*, *33*, *34*). Here, we present the results of our seafloor geodetic response to the 2021 M8.2 Chignik, Alaska, subduction zone earthquake (*35*, *36*).

## RESULTS

### Seafloor geodetic response to the Chignik earthquake

**Fig. 2.**
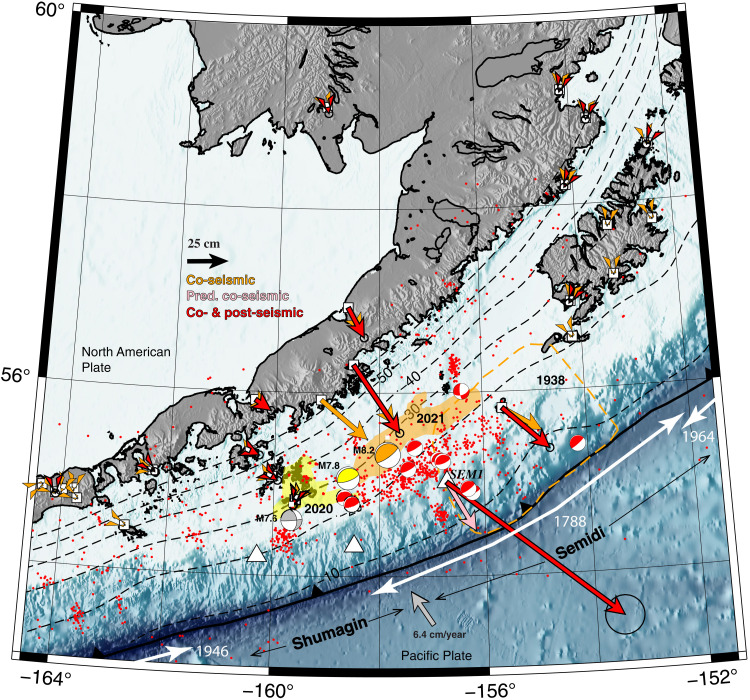
Chignik earthquake location and observations. Topography and bathymetry of the Alaska subduction zone’s Semidi section. Black dashed lines, megathrust depth contours (*85*). Colored regions, rupture patches (~1-m slip contour) of 1938 (orange-dashed) (*42*), Simeonof (2020, yellow) (*37*), and Chignik (2021, orange) (*41*) earthquakes. See Supplementary Text for further discussion of 1938 rupture constraints. Orange beachball, focal mechanism of the Chignik earthquake (*55*); yellow beachball, focal mechanism of the Simeonof earthquake (*93*); gray beachball, focal mechanism of the 2020 Sand Point earthquake (*94*); small red circles, aftershocks of the Chignik earthquake from National Earthquake Information Catalog (https://earthquake.usgs.gov/earthquakes/search) from 29 July 2021 to 12 January 2022; red beachballs, focal mechanisms for Chignik aftershocks from Harvard Centroid Moment Tensor (CMT) catalog (table S3) (*95*, *96*); white triangles, GNSS-A stations; white squares, subaerial continuous GNSS stations. See fig. S1 for GNSS station codes. Orange vectors, Chignik co-seismic displacement; red vectors, Chignik cumulative co- and post-seismic displacements; pink vector, predicted displacement at GNSS-A station SEM1 from the co-seismic model (*40*); white arrows, along-strike rupture extents of substantial recent trench earthquakes; black arrows, boundaries of trench sections.

The 29 July 2021 *M*_w_ (moment magnitude) 8.2 Chignik Alaska, subduction zone earthquake was the largest earthquake within the U.S. territory in 58 years (Fig. 2). The Chignik event was the third in a triad of Alaska subduction zone events that includes the 21 July 2020 *M*_w_ 7.8 Simeonof and the 19 October *M*_w_ 7.6 Sand Point earthquakes (Fig. 2) (*36*–*38*). The Chignik and Simeonof events ruptured the subduction zone megathrust, and the Sand Point event was an intraslab strike-slip event; the first two events ruptured the Shumagin section of the subduction zone, whereas the Chignik earthquake ruptured the Semidi section to the east (*38*). Plate convergence rate at the Semidi section is ~6.3 cm/year, and the slip deficit rate (the amount of plate boundary slip stored elastically in the upper plate) varies west to east from 33 to 100% (*39*). The Chignik event nucleated at ~26 km depth and ruptured up-dip toward the northeast (*35*, *36*, *40*, *41*) and the inferred rupture zone of the 1938 tsunamigenic *M*_w_ 8.2 to 8.3 earthquake (*36*, *42*). Previous analyses suggested that Chignik co-seismic slip was confined below the intermediate depth portion of the megathrust below the continental shelf (~20 to 25 km), consistent with the lack of a large associated tsunami (*36*, *40*–*42*). Liu *et al.* (*36*) assert that the megathrust immediately up-dip of the principal Chignik rupture patch retains a substantial slip deficit (their peak slip was ~8.2 m) that could be recuperated in a future tsunamigenic earthquake or via shallow afterslip. Similarly, Elliot *et al.* (*35*) estimate increased Coulomb failure stress in the shallow portion of the megathrust.

The Semidi section poses substantial Pacific basin-wide tsunami hazards. The 1938 *M*_w_ 8.3 earthquake (Fig. 2) generated a tsunami detected at tide gauges in Honolulu and Southern California (*42*–*44*). The 1788 earthquake (Fig. 2) was likely substantially larger; it caused grave damage to a settlement on Sitniak island where it likely generated a tsunami with wave heights potentially up to 10 m (*43*–*46*). Moreover, a substantial tsunamigenic earthquake on the Semidi section was the scenario event for the USGS Science Application for Risk Reduction (SAFRR) modeling exercise (*47*). Thus, timely assessment of the post-Chignik seismic and tsunamigenic potential of the shallow megathrust may allow near real-time situational awareness of Pacific basin-wide hazards.

We installed and made initial measurements on three ocean-bottom GNSS-Acoustic (GNSS-A) (*29*, *31*, *48*) sites in 2017–2019 spanning portions of the Shumagin and Semidi sections at ocean depths of 1000 to 3000 m (Fig. 2 and table S1). On the basis of the proximity of GNSS-A site SEM1 to the M8.2 epicenter, we chose to mount a post-earthquake remeasurement campaign. From 15 October to 1 November 2021, we resurveyed SEM1 with an autonomous ocean surface vessel (*49*). Despite especially challenging sea conditions (Materials and Methods), we acquired a total of ~20 hours of high-quality GNSS and acoustic transponder data spaced over 2 weeks from which we determine a cumulative co- and post-seismic offset estimate of ~1.4-m and ~1.1-m east and ~83-cm south displacement (see Fig. 2 and Materials and Methods). Station SEM1 is ~60 km from the subduction zone trench, only for the 2011 *M*_w_ 9 Tohoku earthquake has geodetic observations been made closer (~50 and 21 km) to a trench (*8*).

### Cumulative megathrust slip

To interpet SEM1’s displacement, it is important to consider potential contributions from different slip and deformation processes, given that the station was reoccupied ~2.5 months after the M8.2 Chignik mainshock. Regional subaerial continuous GNSS displacements demonstrate post-seismic motions up to 500 km from the Chignik epicenter (Fig. 2, Materials and Methods, and tables S2 and S3). Post- and co-seismic displacement vectors are generally oriented within a few degrees of one another, and comparison of co-seismic to cumulative co- and post-seismic displacement magnitudes demonstrates that the cumulative signal is ~1.2 to 1.5 times the co-seismic (Fig. 2). SEM1’s vector is oriented up-dip and toward the trench. Viscoelastic modeling of asymmetric megathrust rupture demonstrates diagnostic post-seismic landward motion for near-trench locations such as SEM1’s (*50*); the landward-directed post-seismic GNSS-A displacements for the Tohoku earthquake were the principal evidence that viscoelastic relaxation processes dominated the Tohoku post-seismic signal (*51*). The size and extent of the viscoelastic signal for the M8.2 Chignik event are expected to be smaller than for the M9 Tohoku event because of both its narrower down-dip extent and its smaller slip magnitude (by as much as an order of magnitude) (*32*, *51*). The combination of these factors leads us to conclude that SEM1’s trenchward orientation is best explained by some combination of co- and/or post-seismic slip on the main rupture plane or a fault with similar strike and dip-direction. If anything, the landward viscoelastic contribution would diminish the trenchward displacement, making megathrust slip estimates a minimum (*50*). Separating the SEM1 vector into co- and post-seismic components is highly model-dependent and uncertain, however (*26*). Accordingly, we choose the least ambiguous approach and focus our analysis on cumulative co- and post-seismic slip on the megathrust from the time of the earthquake to the time of SEM1’s measurement.

**Fig. 3.**
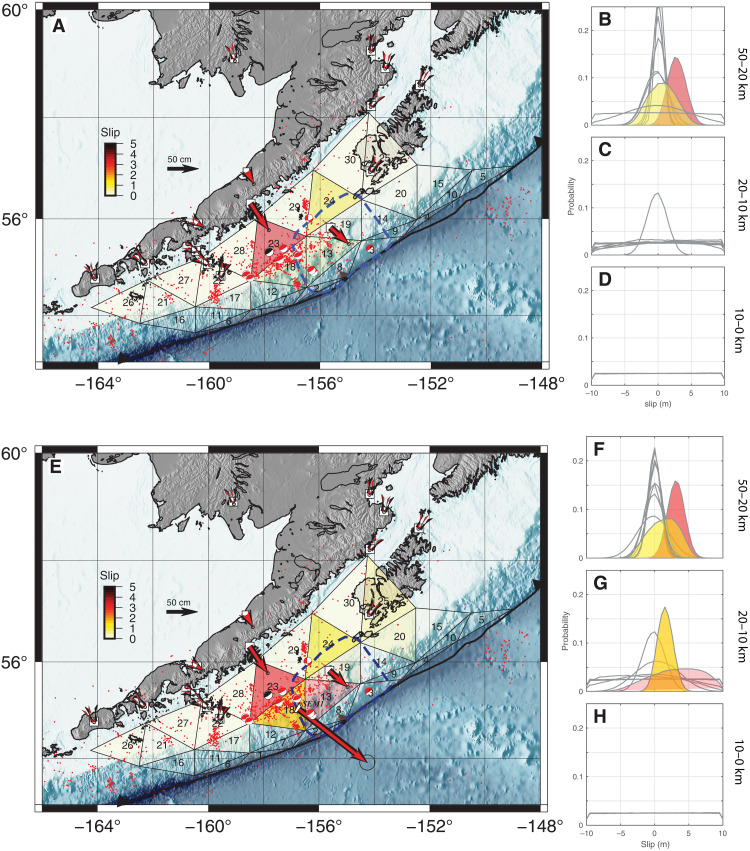
Megathrust cumulative slip models. (**A**) Without GNSS-A constraint. Mean model from MCMC inversion run on cumulative co- and post-seismic displacements, without including GNSS-A station SEM1. Black vectors, cumulative co- and post-seismic displacement; red vectors, displacements predicted from the mean model; small red circles, aftershocks of the Chignik earthquake from National Earthquake Information Catalog (https://earthquake.usgs.gov/earthquakes/search) from 29 July 2021 to 12 January 2022; black beachball, NEIC focal mechanism for the Chignik mainshock (*55*); red beachballs, focal mechanisms for Chignik aftershocks from Harvard CMT catalog (*95*, *96*); black triangles, boundaries of triangular dislocation subfaults. Each subfault is colored by the magnitude of its average slip vector, with transparency scaled by the width of the marginal slip distributions shown in (B) to (D). Index numbers in each subfault are keyed to distributions in (B). (**B** to **D**) Marginal distributions on slip magnitude for each subfault. Distributions with nonzero mean values are colored. (B) Fifty to 20 km depth. The two distributions with colored backgrounds are to aid in visual connection with the indexed subfaults 23 and 24 in (A). (C) Twenty to 10 km depth. (D) Ten to 0 km depth. See fig. S5 for each individual posterior distribution with dip- and strike-slip components. (**E** to **H**) Same as (A) to (D) except with GNSS-A station SEM1 included in the MCMC inversion. (F) Fifty to 20 km depth. The two distributions with colored backgrounds are to aid in visual connection with the indexed subfaults 23 and 24 in (E). (G) Twenty to 10 km depth. The two distributions with colored backgrounds are to aid in visual connection with the indexed subfaults 13 and 18 in (E). Ten to 0 km depth. See fig. S6 for each individual posterior distribution with dip- and strike-slip components.

**Fig. 4.**
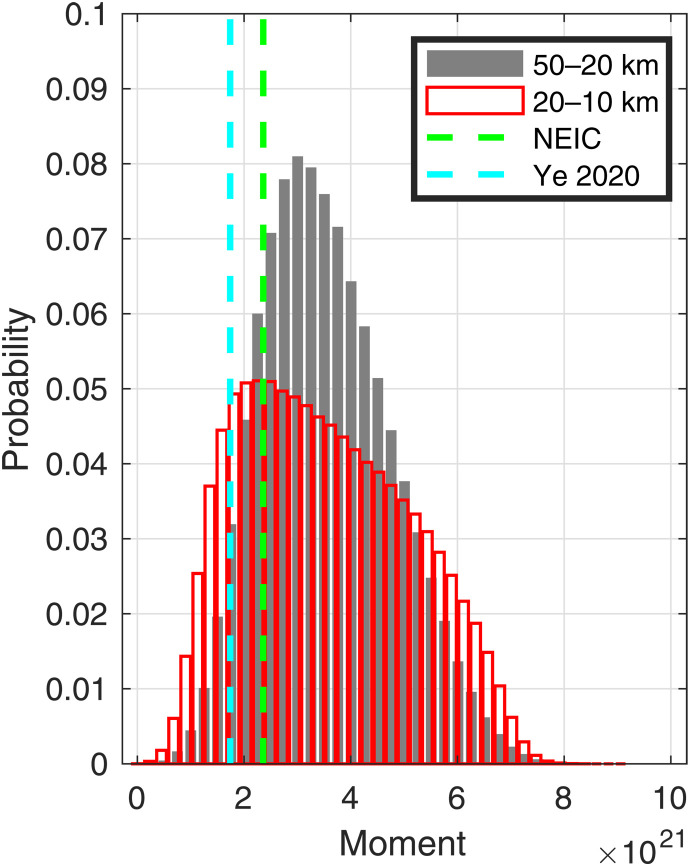
Down- and up-dip moment. Posterior distribution for seismic moment summed over deeper (50 to 20 km) and shallower (20 to 10 km) subfaults. Green dashed line is the estimated seismic moment, and cyan dashed line is from Ye *et al.* (*41*).

The principal objective of our analysis is to use the GNSS-A displacement to constrain the relative proportion of down- and up-dip cumulative slip on the megathrust, and it is complementary to previous work that constrained the details of the rupture process (*35*, *36*, *40*, *41*) and the up-dip limit of co-seismic slip to 20 to 25 km depth (*40*, *41*). We show by considering the individual components of the National Earthquake Information Center (NEIC) joint seismogeodetic inversion that the land-based GNSS data alone adequately constrain the spatial slip boundaries (see the Supplementary Materials). This permits us to design a computationally efficient analysis that uses only static displacements and Markov chain Monte Carlo (MCMC) sampling (*52*, *53*) to thoroughly explore model parameter space to assess the uncertainty and resolution of the multitude of possible slip models. To honor both the changes in dip and the along-strike curvature of the megathrust, we use triangular dislocations in an elastic half space (see Fig. 3 and the Supplementary Materials). The vertices of the triangular subfaults in our mesh are fixed to the 50-, 20-, and 10-km contours, respectively, of the megathrust (*19*), and we permit (but do not require) slip to the trench (Fig. 3). We do not impose any smoothing of slip across the subfaults, they are free to slip independently of one another, and we use a method that explicitly considers the relation between subfault size and the resolving power of the geodetic network (*54*) to arrive at subfault sizes with area an order of magnitude greater than the previous models (see Figs. 3 and 4 and Materials and Methods). Hence, our analysis is designed to constrain average, rather than peak, slip on the megathrust.

We perform two inversions differing only in the inclusion of the displacement from station SEM1 (Fig. 3). For each inversion, we show the mean model (Fig. 3, A and E) and the posterior marginal slip distributions (Fig. 3, B to D and F to H) for 30 triangular subfaults from more than one million forward model evaluations. The mean model is a good indicator of a preferred model, whereas the width of the marginal distributions reflects model resolution. We stress that the mean model should only be interpreted in the resolution context provided by the marginal distributions and, accordingly, we scale the transparency of each subfault’s mean slip value (Fig. 3, A and E) by the width of its associated marginal distribution (Fig. 3, B to D and F to H).

In general, there are three classes of marginal distributions: well-constrained (normally distributed) with nonzero mean values (Fig. 3A, subfaults 23 and 24; Fig. 3E, subfaults 13, 18, 23, and 24); well-constrained with roughly zero-centered mean values (e.g., Fig. 3, A and E, subfaults 26 to 30); and unconstrained (uniformly distributed, the prior constraint; e.g., Fig. 3, A and E, subfaults 1 to 5). Only deeper slip is constrained in the model without station SEM1 (Fig. 3A); the two subfaults that are well constrained with nonzero mean values are both deeper than 20 km with slip values ranging from ~1 to 3 m. Shallower than 20 km, slip values are well constrained in only two subfaults, and each of these has a mean slip of zero. This result is spatially similar to the previously published models that all used the subaerial GNSS data sets, although our analysis has maximum slip values of ~3 m compared with ~5 to 10 m for the other analyses (*35*, *36*, *40*). In contrast, up-dip slip is constrained by the model that includes SEM1 (Fig. 3, E to H). This model has two well-constrained nonzero mean marginal distributions with a slip of ~1 to 3 m shallower than 20 km, located immediately up-dip and east of the Chignik hypocenter. The inclusion of the GNSS-A station permits the resolution of up-dip slip that is not resolvable with only land-based observations.

Deeper and shallower cumulative seismic moments (*M*_0_ *=* μ*As*; where μ shear modulus is taken to be 30 GPa, *A* is the subfault area, and *s* is the average subfault slip) calculated from our analysis are distributed similarly and compare favorably with the NEIC’s (*55*) and the independent teleseismic moment estimates for the Chignik event of Ye *et al*. (*41*) (Fig. 4). We suspect that the somewhat higher mean cumulative moment estimate for the deeper portion of the megathrust is due to ongoing down-dip afterslip (Fig. 2). The similar distributions of down- and up-dip moment are an important constraint that follows, in part, from our conservative use of subfaults that reflect average slip over relatively larger areas. Even if there were strongly variable co-seismic slip at depth, producing greater peak slip values over localized patches (*35*, *36*, *40*, *41*), our results show that on average deeper and shallower moments are equilibrated.

The moment equivalence strongly suggests that the shallower slip is post-seismic afterslip rather than co-seismic slip: If the deeper and shallower moment both resulted from co-seismic slip, then the summed moment estimate would be roughly double the independent teleseismic co-seismic moment estimates (Fig. 4). This conclusion is consistent with the seismogeodetic models including tsunami data that require up-dip co-seismic slip to reach no shallower than 20 km (*40*, *41*). Although modeling of tsunami data is dependent on mechanical assumptions, the possible activation of splay faults (*4*, *56*–*59*), and sometimes poorly known bathmetry near water-level sensors, inclusion of tsunami data seaward of recent megathrust earthquakes has been shown to help constrain up-dip co-seismic rupture extent (*60*–*65*). Last, the along-strike–trending band of aftershocks near the 20-km contour is also consistent with a stress concentration arising from a displacement gradient at the up-dip limit of co-seismic slip at ~20 km depth (Fig. 2) (*66*). Although depth constraints for these events are poor, the available focal mechanisms are oriented along-strike with a shallow dipping focal plane that is also consistent with megathrust interface slip (Fig. 2). If the aftershocks were in the underlying plate, we would expect much more variability in faulting style, including strike-slip and/or normal mechanisms (*67*). Thus, by ~2.5 months after the main shock, it appears that slip on the up-dip portion of the megathrust had effectively equilibrated with the down-dip portion, diminishing the potential slip deficit. Further, up-dip afterslip appears to have been dominantly aseismic: Our estimate of summed seismic moment of the Chignik aftershocks ranges from 2.8 × 10^19^ to 2.5 × 10^20^ Nm (Fig. 2 and table S4), which is ~1 to 10% of the mean value (~2 × 10^21^ Nm) of the estimated up-dip post-seismic moment.

## DISCUSSION

**Fig. 5.**
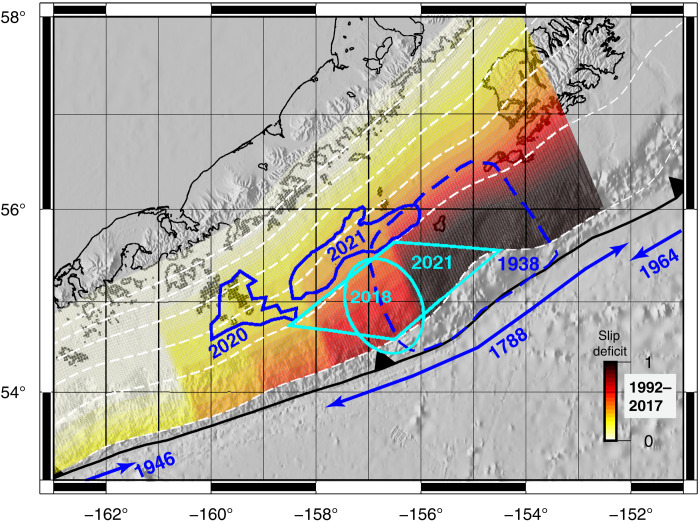
Compilation of the boundaries and types of slip events on the megathrust for the Semidi section from 1938 to the present. Dark blue indicates co-seismic slip; cyan indicates aseismic slip. 1938 earthquake (*42*). 1992 to 2017 interseismic slip deficit (*39*), 0 indicates continuous slip at the convergence rate of 6.3 cm/year, 1 indicates no slip on the megathrust interface. 2018 SSE (*68*). 2020 M7.8 Simeonof earthquake (*37*). 2021 Chignik earthquake (*40*). The spatial extent of 2021 Chignik earthquake up-dip afterslip is taken from the triangle borders of subfault indices 13 and 18 from Fig. 3B.

Our observations and analysis, combined with the instrumental and observational history of the region, permit placing rare process and hazards constraints on the up-dip, potentially tsunamigenic, portions of a subduction zone megathrust. In Fig. 5, we compile the spatial limits of all slip events constrained for the past 83 years on the Semidi section of the megathrust. Co-seismic tsunamigenic slip in 1938 extended from ~30 km depth to shallow depths, perhaps reaching close to the seafloor (*42*) (see also Supplementary Text, “Uncertainty associated with the 1938 event and rupture limits”). From 1992 to 2017, stable sliding and slip rate deficit on the megathrust graded eastward into a fully kinematically coupled portion that coincided with the 1938 rupture zone (*39*). Recently, ocean-bottom pressure sensor data permitted the inference of an accelerated intermediate-shallow depth slow-slip event (SSE) in 2018 (*68*). The 2020 Simeonof earthquake produced co-seismic slip that did not extend shallower than ~20 km (*37*). Similarly, the deep co-seismic slip from the July 2021 Chignik did not extend up-dip further than ~20 km depth (*40*). Last, as we report above, up-dip afterslip following the Chignik mainshock continued through October 2021.

An important realization that emerges from this compilation is that the Chignik up-dip afterslip occurred on portions of the megathrust at the same depth that had previously experienced end-member slip styles—stable sliding and dynamic rupture. To the best of our knowledge, only for the Tohoku event, where the co-seismic slip overlapped with a previously inferred SSE zone (*15*), and for the 2015 Illapel, Chile event, where the afterslip was inferred on the margins of the co-seismic slip distribution (*18*), have there been similar inference for megathrust slip behavior. For the Chignik event, the western afterslip portion occurred where a previous stable slip in the form of interseismic creep and a SSE had occurred (*39*, *68*). In contrast, the eastern afterslip portion overlapped with an up-dip portion of the megathrust inferred to have ruptured both co-seismically in 1938 (*42*) and to have recently exhibited lack of a slip rate deficit and full kinematic coupling (*39*). We note that the post-Chignik afterslip zone overlaps the 1938 rupture zone even with generous uncertainties on its limits (see the Supplementary Materials). For instance, because our model has no resolving power at depths shallower than 10 km, whether the 1938 event ruptured shallower than 10 km depth is immaterial to the conclusion that there is a notable overlap between it and the Chignik up-dip afterslip. Thus, if slip style is reflective of fault plane frictional character, then the stable sliding of the western afterslipping portion is consistent with inferred velocity-strengthening character (*69*) and the long-held mechanical generalization that up-dip portions of megathrusts are VS (*10*). However, the extension of afterslip into the eastern portion, which exhibited typical VW character associated with dynamic rupture in 1938 and recent full kinematic coupling, indicates variable frictional behavior.

We reconcile the eastern afterslipping observations with possible theoretical explanations that permit shallower megathrust regions to occasionally experience dynamic rupture while generally maintaining VS character. One possibility is that the intrinsic material rate-dependent frictional property of the Semidi shallow megathrust, modified by varying topographic roughness of the down-going plate (*70*–*73*), is conditionally stable rather than strict VW or strengthening (*69*, *74*, *75*). To explain the Tohoku observations, for instance, Ito *et al.* (*14*) suggested that conditionally stable frictional properties of clay-rich fault gouge could lead to weak VW character at low slip rates and strong VW character at high slip rates as demonstrated in laboratory materials (*58*). A second possibility is the theoretical suggestion that co-seismic slip can extend into VS zones from processes such as reflections of seismic waves off the seafloor interface, causing stress changes on the megathrust (*16*), or elevated pore-fluid pressures leading to thermal pressurization and megathrust dynamic weakening (*17*). This is consistent with the Semidi section being characterized by a thick pile of down-going sediment (*73*) and by elevated fluid pressurization (*72*). In this case, given VS frictional character, we might expect the megathrust to exhibit interseismic creep, which is contrary to the inferred high slip rate deficit (Fig. 5) (*39*). A final possibility that would permit VS character without interseismic creep is that a shallower megathrust portion could have high kinematic coupling (a high slip rate deficit) and be frictionally uncoupled due to a deeper locked patch and an up-dip stress shadow zone (*2*); the shallower portion would be free to slip in response to applied shear stress (*25*) such as imparted from the deeper Chignik rupture (*35*, *36*). The fully coupled down-dip portion of the 1938 rupture zone could be the source of a potential stress shadow (*2*). Without much denser observational networks and ground truthing of mechanical properties at the megathrust interface from, for instance, drilling and in situ measurements (*76*), it may not be currently possible to favor one of these end-member theoretical explanations. This could be further complicated by spatial frictional variability on the shallower megathrust over spatial scales shorter than the length of the Semidi section. Nonetheless, our analysis of Chignik afterslip does not refute the notion that the up-dip portion of the megathrust can exhibit general VS character (*10*).

We have shown that, as anticipated (*77*), seafloor geodetic observations definitively increase the resolving power of slip inferred on megathrusts (Fig. 3); this, in turn, permits modifications to time-dependent hazards inferences. For instance, the inclusion of a single seafloor geodetic station into our analysis allows us to conclude that the slip deficit on the up-dip portion of the megathrust resulting from down-dip Chignik co-seismic slip was rapidly and substantially diminished by rapid afterslip. Accordingly, because down- and up-dip slip have been roughly equilibrated, the lack of a slip gradient makes it unlikely that a corresponding stress gradient persists (*35*, *78*, *79*), at least for depths greater than ~10 km where our model has resolution (Fig. 3). It appears that for the Semidi section, a sizeable (M8.2) earthquake temporarily perturbed the state of slip and stress on the megathrust, but it probably did not leave the shallower portion of the megathrust in any more of a primed state to slip in a major trench-rupturing Pacific basin-wide tsunamigenic earthquake than before (*80*, *81*). Although some of the long-term slip-deficit in the Semidi section (*39*) was recovered by the Chignik event, it is clear that the potential for a much larger, 1788-type earthquake still dominates the hazard for the Semidi section (*39*, *43*, *46*, *47*).

Rapid seafloor geodetic response and attendant analysis of the state of slip on a portion of a shallow megathrust could be replicated and refined for other similarly hazardous subduction zones. Moreover, mobilization and deployment of autonomous surface vessels do not require large oceanographic research vessels and so response times could certainly be reduced to days or hours. This could also motivate construction of seafloor geodetic networks to establish pre-event baselines. Even with these seafloor geodetic measurements in Alaska, some of the closest ever made to the trench, our observations are still ~60 km from the trench itself. The technical capability exists to make seafloor geodetic measurements at the deeper water depths where megathrusts reach closest to the seafloor and these regions could be prioritized in future observational efforts.

## MATERIALS AND METHODS

### Seafloor geodetic mission, data, displacement estimate

The GNSS-A method for seafloor positioning with precision adequate for geophysical applications such as seismo- or volcano-tectonic studies was developed over the past ~3 decades (*29*, *31*, *34*, *48*, *51*, *82*, *83*). We refer readers to two review papers for thorough overviews of the technique and its application (*82*, *83*). The basic components of our processing have been documented previously (*29*, *31*, *48*) and, here, we describe the pertinent aspects of our analysis of station SEM1 before and after the Chignik earthquake.

In general, GNSS-A consists of an array of acoustic transponders deployed on the seafloor that is surveyed by a platform on the sea surface that ranges the transponders acoustically from the center of the array. By surveying each transponder simultaneously from the array center, we can position the array relative to the surface platform; the entire system is then anchored in a terrestrial reference frame using GNSS on the surface platform (*31*). The SEM1 array surveyed in this study consists of three transponders, which is the minimum number of transponders required to resolve the horizontal position of an array. These transponders are deployed on permanent concrete benchmarks arranged in a concentric circle with radius approximately equal to the seafloor depth (in this case, approximately 1200 m). The transponders were recovered in August 2022 at the end of their battery lifetimes but can be reinstalled onto the benchmarks using a remotely operated vehicle (ROV) to extend the observations anytime in the future. For our surveys, we used an autonomous “Wave Glider” surface vessel.

We determine the Wave Glider’s position using the Gipsy-X software (*8*). We model the two-way travel time from the Wave Glider to each transponder following Chadwell and Sweeney (*29*), with the raytrace inversion constrained by a conductivity-temperature-depth profile (*29*). We refer to the difference between this modeled two-way travel time and the two-way travel time we measure in the field as the GNSS-A residual. We use the GNSS-A residuals as input data for a weighted linear least squares inversion to solve for the best-fitting horizontal offset of the transponder array, assuming that the array moves as a block. In this inversion, the weights are a linear combination of the instrument error of the acoustic transducer mounted on the underside of the Wave Glider and the propagated formal GNSS errors of the Wave Glider positions. Although the array only has three transponders and we are solving for four parameters (three position parameters and the nadir total delay of the sound velocity profile), the variation in sound velocity will map into the vertical component as long as the water column approximates a horizontally layered medium (*29*, *31*) because we survey from the array center and treat the array as a block. Biases in the array position are minimized by flagging and removing epochs when the Wave Glider was further than 150 m from the array center or when the GNSS-A residuals were larger than normal, which is usually a sign of noise contamination due to factors such as GNSS cycle slips.

Station SEM1 was surveyed during three missions: (i) 13 May to 28 May 2018, (ii) 2 June to 15 June 2019, (iii) 1 October to 15 October 2021(table S1). Figure S2 shows an overview of the GNSS-A residuals for each of the three surveys at SEM1. In this figure, we averaged the residuals from the three transponders in the array and rotated them into a local East-North frame to present a clearer picture of the apparent motion of the array we witnessed throughout the surveys. The spatial scatter is a good assessment of the positional uncertainty, and the offset observed after the Chignik earthquake is readily apparent. The residuals indicate a repeatability at two SDs (2σ) of better than 5 cm, which is fairly standard for the GNSS-A technique.

To estimate the cumulative co- and post-seismic offset due to the Chignik event, we fit a linear trend to the two pre-event locations, project that to the date of the earthquake, and then calculate an offset (fig. S3). The 2019 position has a 1σ uncertainty of 0.2 cm in the east component and 0.22 cm in the north component. For the 2021 position, these numbers are 0.61 cm in east and 0.65 cm in north. Thus, a 2σ SD for the offset between these two is ~1.3 to 1.4 cm.

### Subaerial GNSS displacements

For co-seismic displacements, we use the GNSS data and offsets produced by the Nevada Geodetic Laboratory (Fig. 2 and table S3; http://geodesy.unr.edu/news_items/20210730) (*84*). To estimate cumulative co- and post-seismic displacements, we use the daily GNSS time series product produced (24-hour final solution in IGS14 reference frame) by the Nevada Geodetic Laboratory (Fig. 2 and fig. S1; http://geodesy.unr.edu) (*84*). For each coordinate (East, North, Up), we find the pre-event displacement by linearly regressing the daily displacements for the week before the earthquake (not including the day of the event). We find the post-event displacement by taking the mean value of the daily displacements during the time period of the GNSS-A measurements (1 to 16 October 2021). The cumulative co- and post-seismic displacement is then the difference of the post- and pre-event displacements.

### Statistical analysis

#### NEIC seismogeodetic finite fault inversion

The USGS NEIC creates FFMs using teleseismic broadband data and available regional accelerometer and geodetic observations (*24*, *85*) to generate event web pages that provide a suite of earthquake source information from the moment a notable event happens. The FFM is generated using a nonlinear inversion approach to modeling slip amplitude, rake, rupture time, and rise time on a discretized fault plane made up of smaller subfaults, finding the solution that best fits the observations in the wavelet domain. The nonlinear inversion is based on the work of Ji *et al.* (*23*).

Figure S4 shows the combined FFM (fig. S4A) and individual component models computed with teleseismic broadband (fig. S4B), regional strong-motion accelerometer (fig. S4C), high-rate GNSS (fig. S4D), and static GNSS observations (fig. S4E). All data used in the inversions have been previously published (*24*) and are publicly available (*86*). The intent of this exercise is to show the spatial resolving power of each component of the inversion and so this example does not include GNSS-A station SEM1. The principal spatial character of the inverted slip patch (along-strike and down-dip dimensions) are equivalently resolved by the teleseismic (fig. S4B) and GNSS (fig. S4E) components of the solution, with much smaller contributions from the single regional strong motion accelerometer station (fig. S4C) and the high-rate GNSS stations (fig. S4D). This shows that an analysis using geodetic data alone is adequate to characterize the average slip magnitude on the megathrust.

#### Subfault size determination

We use a formal process of optimal triangular subpatch sizing (*54*) to arrive at a more appropriate triangle size for the cumulative co- and post-seismic Chignik inversion. The Barnhart and Lohman (*54*) optimization technique relies on all triangles to be coplanar with one another. In our final inversion design (Fig. 3), we use the basic triangle size from the Barnhart and Lohman (*54*) triangle optimization inversion and tie the triangle vertices to the contours of the Slab2 dataset (*19*).

#### Gibbs sampling/MCMC slip analysis

We construct a forward model by precomputing Green’s functions for surface displacements arising from slip across dislocations in an elastic half-space for triangular subfaults (*87*–*89*) defined to be fixed to the Slab2 (*19*) 10-, 20-, and 50-km contours, respectively. Because our observation locations are on the continental shelf, our analysis assumes a flat Earth and topographic effects are not modeled (*15*, *90*). We then use the Gibbs sampling method (*52*) to carry out a thorough MCMC sampling of the parameter space. The misfit (or “energy” in the Gibbs sampling algorithm) is the L2 norm between data and model.

We assign conservative uncertainties of ±1 cm horizontal ±3 cm vertical for the on-land GNSS displacements and ±5 cm for the (*52*) GNSS-A horizontal displacement, although its formal errors are smaller (0.2 to 0.6 cm SD as described above). We do this in an attempt to mitigate model prediction errors—unmodeled errors in the elastic structure, fault geometry, etc. which are out of the scope of this work to address (*53*, *91*). Because unmodeled errors contribute to both GNSS and GNSS-A prediction errors, we assign uncertainities to each of these data types that are higher than their formal errors. We scale the GNSS-A uncertainty more than the GNSS sites because its model prediction error will be greater than the GNSS sites due to its greater magnitude displacement (*53*). We show the marginal distributions for both dip-slip and strike-slip components from the inversion without (fig. S5) and with (fig. S6) GNSS-A station SEM1.

#### Aftershock seismic moment sum

To assess how much up-dip slip was seismically accommodated, we sum the moments of Chignik aftershocks in the NEIC catalog defined as occurring between the time of the main event (29 July 2021) until the last day of GNSS-A surveying of site SEM1 (1 November 2021) and with epicenters in subfaults 2, 3, 8, 12 to 14, 18, 19, 23, and 24 (Fig. 3E and table S3). The catalog reports *M*_w_ and we convert that to moment, *M*_0_ (units of Newton meter), using *M*_0_ *=* 10^(1.5*M_w_ *+* 16.1)^ *M*_0_ *=* 10^(1.5*M_w_ *+* 9.05)^ (*92*) to find a range of summed values of 2.8 × 10^19^ Nm to 2.5 × 10^20^ Nm. This value is likely a conservative, upper limit on the seismic component of up-dip afterslip because of our somewhat overly broad definition of up-dip Chignik aftershocks that includes some events that occurred in the down-dip rupture zone of the Chignik mainshock (Fig. 3E).

## Supplementary Materials

### This PDF file includes:

Supplementary Text Figs. S1 to S6 Tables S1 to S4 References
