# Supplementary material for: Rapid shallow megathrust afterslip from the 2021 M8.2 Chignik, Alaska earthquake revealed by seafloor geodesy
Source: Sci Adv. 2023 Apr 26;9(17):eadf9299. doi: 10.1126/sciadv.adf9299 (PMC10132754; doi:10.1126/sciadv.adf9299)
Supplement: Supplementary file 1 — Supplementary Text Figs. S1 to S6 Tables S1 to S4 References [file sciadv.adf9299_sm.pdf]

Science Advances
AAAS

Supplementary Materials for

Rapid shallow megathrust afterslip from the 2021 M8.2 Chignik, Alaska earthquake revealed by seafloor geodesy

Benjamin A. Brooks *et al.*

Corresponding author: Benjamin A. Brooks, bbrooks@usgs.gov

Sci. Adv. **9**, eadf9299 (2023)
DOI: 10.1126/sciadv.adf9299

This PDF file includes:

Supplementary Text
Figs. S1 to S6
Tables S1 to S4
References

Supplementary Text

Uncertainty Associated with the 1938 Event and Rupture Limits

We note that, to be expected with attempted models of a historical event, there is considerable uncertainty regarding the 1938 earthquake rupture zone and its associated tsunami. Freymueller et al. (*42*) considered initially a 3x3 grid of rupture models, with slip concentrated either at shallow, middle or deep depths, and western, central or eastern along strike. The model rupture areas were large in all cases, so that peak slip was only a few meters, and there was considerable overlap in the rupture areas of the different test models. Nevertheless, the available digitized records with precise timing were clearly better fit by the shallow eastern model, and fit even better by a shallow far eastern model in which the slip was shifted even further to the east. Freymueller et al. (*42*) ultimately found that the shallow eastern and shallow far eastern models were both acceptable fits to the data, which means that any linear combination of the two models with the same moment would also be acceptable. That means a substantial along-strike uncertainty in the likely rupture limits. While the shallow models had the slip peak at shallow depth, the modeling did not rule out the existence of some slip at greater depths – additional modeling work using more spatially concentrated sources may allow such an assessment. We note that none of these uncertainties affect our main conclusion, that it is most likely that a portion of the Semidi section megathrust that we determine experienced post-Chignik afterslip also experienced co-seismic slip in the 1938 earthquake.

Additionally, Liu et al. (*36*) make the qualitative point that the most accurate 1938 model must also produce a modest far-field tsunami. Although it was not the aim of their modeling, the Freymueller et al. (*42*) model produced 20 cm peak to trough waves at Sitka, so it was still a rather weak far-field tsunami. It is worth noting here that although the test models had their peak

slip at shallow depths, the peak slip was not especially large (smaller than that observed for the Chignik event); just because an earthquake has slip to very shallow depth does not automatically mean that the tsunami will be large, if the slip itself is not large.

Freymueller et al. (*42*) also noted other limitations to their modeling approach, which may have resulted from the lack of high-resolution bathymetry at Chirikof Island. There, Nelson et al. (*97*) using stratigraphic records, infer ~10 meters of runup for the 1938 event, which is not explained by the Freymueller model, at least not given the available bathymetric grid resolution. Freymueller et al. (*42*) found that a model that predicted ~10 m runup at Chirikof would overpredict the instrumental observations by a factor of ~2. It is possible that this conflict in observations could be resolved by local bathymetric amplification of tsunami waves at Chirikof, although that explanation is speculative. We note, however, that the anomalously high run-up at Chirikof also could be explained by more local point sources (such as landslides) or spatially confined high slip patches. Moreover, the Chirikof record appears to record many more tsunami than the larger, more regional events recorded at other Aleutian sites.

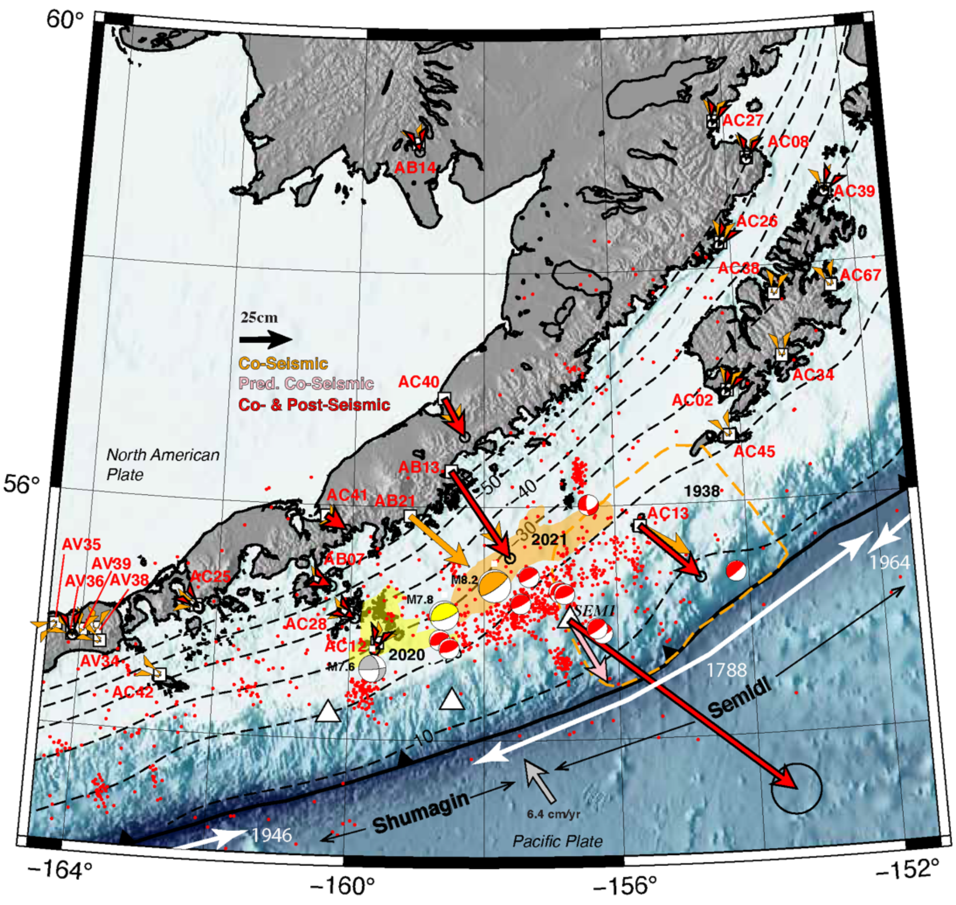

Figure S1. Chignik earthquake location and observations. Same as Figure 2 except with 4-letter GNSS station codes added, red text. Topography and bathymetry of the Alaska subduction zone's Semidi section. Black dashed lines, megathrust depth contours (*85*). Colored regions, rupture patches (~1 m slip contour) of 1938 (orange-dashed) (*42*), Simeonof (2020, yellow) (*37*), and Chignik (2021, orange) (*41*) earthquakes. Orange beachball, focal mechanism of Chignik earthquake (*55*). Yellow beachball, focal mechanism of Simeonof earthquake (*93*). Grey beachball, focal mechanism of 2020 Sand Point earthquake (*94*). Small red circles, aftershocks of the Chignik earthquake from NEIC catalog from 29 July 2021 to 12 January 2022. Red beachballs, focal mechanisms for Chignik aftershocks from Harvard CMT catalog (*95, 96*) (Table S3). White triangles, GNSS-A stations. White squares, subaerial continuous GNSS stations. Orange vectors, Chignik co-seismic displacement. Red vectors, Chignik cumulative co- and post-

seismic displacements. Pink vector, predicted displacement at GNSS-A station SEM1 from co-seismic model (*40*). White arrows, along-strike rupture extents of significant recent trench earthquakes. Black arrows, boundaries of trench sections.

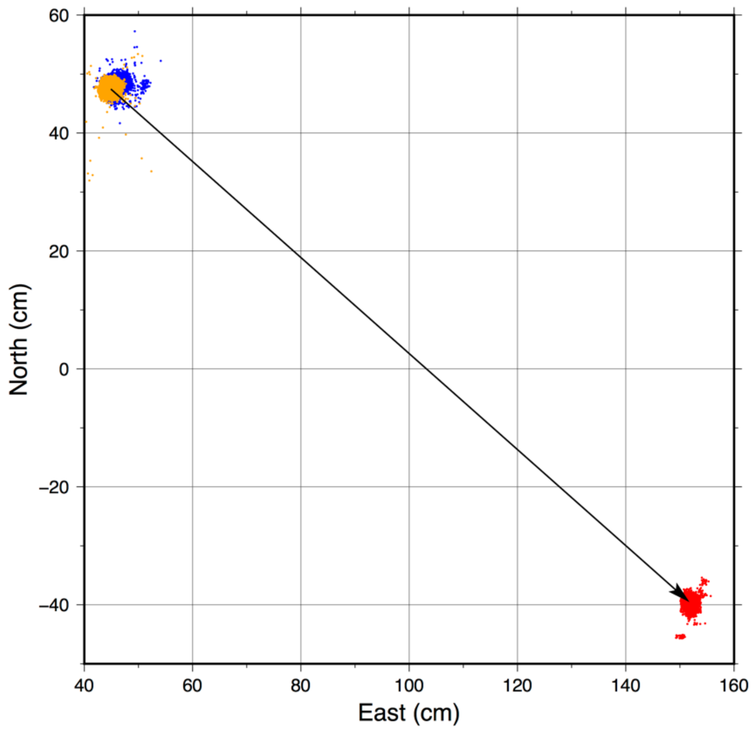

Figure S2. GNSS-Acoustic residuals observed at SEM1 from 2018-2021, rotated into a local East-North frame with the origin equal to the a priori array position. Blue residuals are from the 2018 survey, orange residuals are from the 2019 survey, and red residuals are from the 2021 survey. The black vector denotes the observed offset between 2019 and 2021, primarily due to the Chignik earthquake.

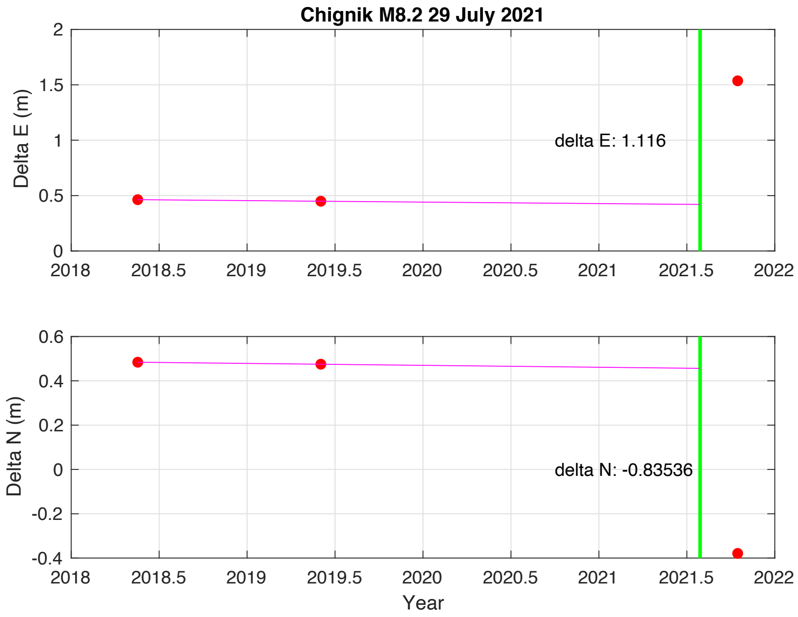

Figure S3. Estimation of cumulative offset for station SEM1 from its *a priori* position. The magenta line denotes the observed array velocity prior to the Chignik earthquake, the occurrence of which is denoted by the green line.

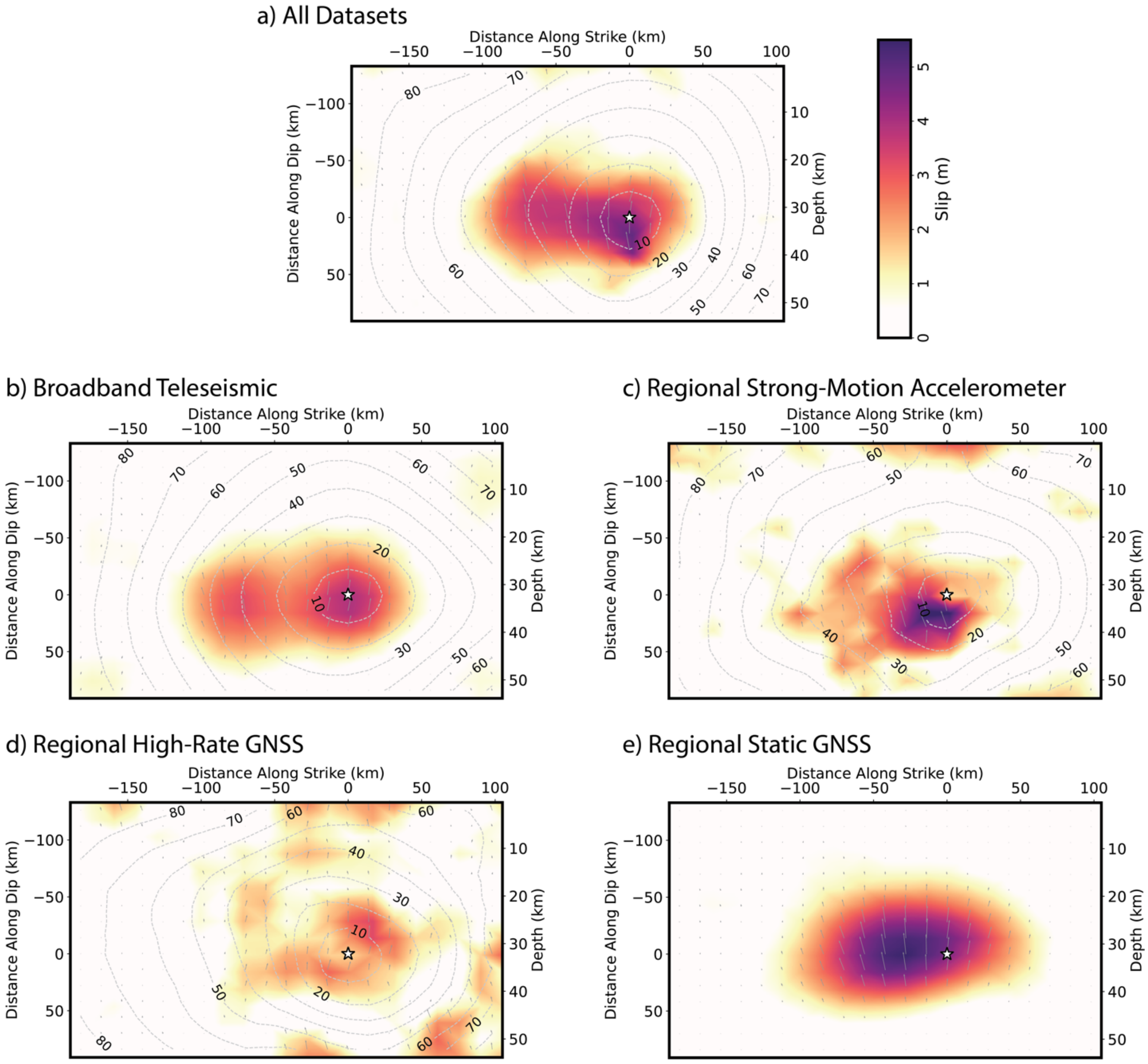

Figure S4. NEIC seismogeodetic finite fault slip inversion for the Chignik earthquake. (A) The composite slip model using all data sets. (B) Broadband teleseismic component. (C) Regional strong-motion accleromter component (1 station). (D) Regional high-rate GNSS displacements. (E) Regional static GNSS component.

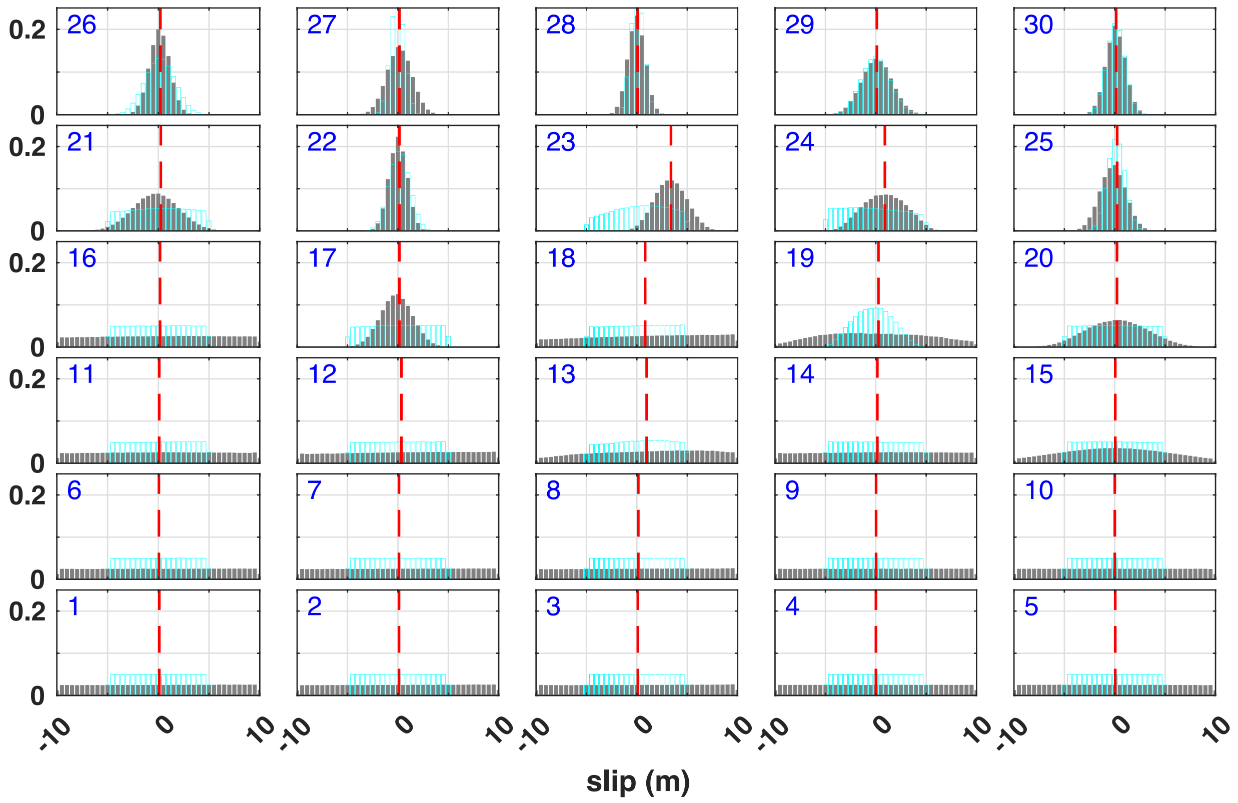

Figure S5. Marginal distributions on dip-slip (grey) and strike-slip (cyan) components for each sub-fault for the inversion that does not include station SEM1. Vertical red dashed line is the mean total slip (dip-slip and strike-slip) value for each sub-fault. Index numbers in each sub-fault are keyed to the triangular sub-faults in Figure 3a.

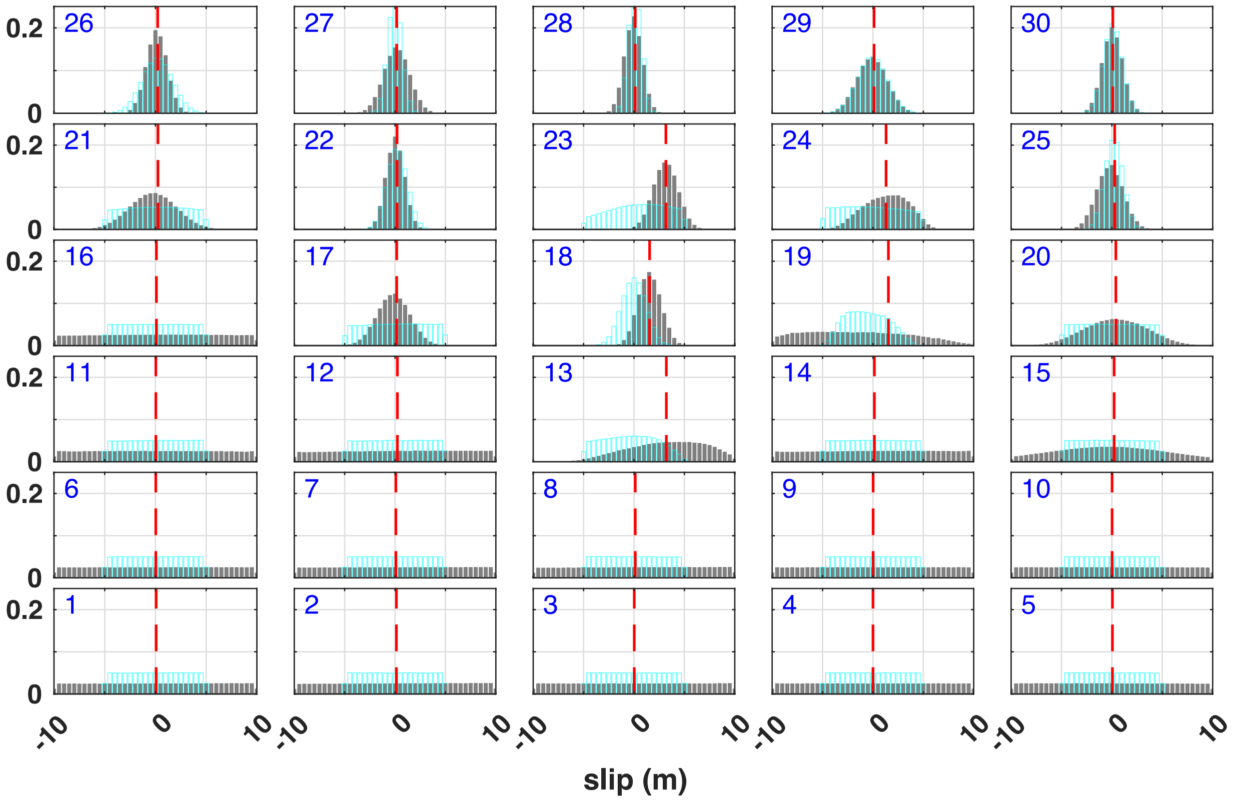

Figure S6. Marginal distributions on dip-slip (grey) and strike-slip (cyan) components for each sub-fault for the inversion that includes station SEM1. Vertical red dashed line is the mean total slip (dip-slip and strike-slip) value for each sub-fault. Index numbers in each sub-fault are keyed to the triangular sub-faults in Figure 4a.

Date	***East displacement (m)***	***North displacement (m)***
5/18/18	0.463	0.484
6/2/19	0.449	0.475
10/15/21	1.51753	-0.39443

Table S1.

Displacements from *a priori* position estimated for GNSS-A stations SEM1.

Station	Lon. (deg)	Lat. (deg)	E (m)	N (m)	U (m)	std E (m)	std N (m)	std U (m)
AB07	-160.4768	55.3493	0.04926	-0.01478	-0.01188	0.00145	0.00221	0.00696
AB13	-158.5038	56.3073	0.25591	-0.3692	-0.07271	0.00145	0.00225	0.00704
AB14	-159.0915	59.1082	0.00953	-0.022	0.0013	0.00149	0.00231	0.00654
AC02	-154.1831	56.9506	-0.0021	-0.00795	-0.00778	0.00146	0.00226	0.00692
AC08	-153.6447	58.9288	-0.00037	-0.00421	0.00019	0.00144	0.00225	0.00612
AC10	-164.8867	54.5226	0.00204	0.00093	0.00008	0.00145	0.00216	0.00609
AC12	-159.5896	54.831	-0.01438	-0.02158	-0.0155	0.00145	0.00219	0.00695
AC13	-155.6224	55.8219	0.24398	-0.14694	0.06159	0.00154	0.0024	0.00765
AC21	-159.1277	55.9211	0.29113	-0.24312	-0.0788	0.00145	0.00223	0.00698
AC25	-162.3141	55.089	0.01087	-0.00208	-0.00251	0.00145	0.00218	0.00677
AC26	-154.1503	58.2146	-0.00194	-0.00943	-0.00111	0.0015	0.00233	0.00674
AC27	-154.1629	59.2525	-0.00078	-0.00716	-0.00355	0.00145	0.00225	0.00607
AC28	-160.0492	55.0785	0.03098	-0.00902	-0.01734	0.00145	0.0022	0.00696
AC34	-153.2792	57.22	0.00009	-0.00289	-0.00264	0.00145	0.00226	0.00672
AC38	-153.3419	57.7537	-0.00122	-0.00515	-0.00362	0.00154	0.00238	0.007
AC39	-152.3941	58.6097	0.00027	-0.00193	-0.00047	0.00145	0.00224	0.00599
AC40	-158.6186	56.9303	0.0863	-0.15232	-0.00385	0.00146	0.00227	0.00707
AC41	-160.4073	55.9087	0.08986	-0.0475	-0.01036	0.00146	0.00226	0.0071
AC42	-162.7836	54.4718	0.00293	-0.00109	-0.00315	0.00144	0.00216	0.00664
AC45	-154.181	56.5645	0.00069	-0.00142	-0.01315	0.00146	0.00226	0.00695
AC67	-152.4254	57.7907	0.00038	-0.00136	-0.00072	0.00144	0.00226	0.00637
AV24	-164.7548	54.59	0.00499	0.00276	-0.00055	0.00147	0.00219	0.00624
AV25	-164.7795	54.53	0.00189	0.00146	0.00189	0.00146	0.00217	0.00618
AV26	-164.5805	54.5716	-0.00224	-0.01194	-0.01053	0.00147	0.0022	0.00633

AV29	-164.5862	54.4723	0.00335	0.00052	0.00064	0.00145	0.00215	0.00618
AV34	-163.7129	54.7249	0.00448	-0.00224	0.00099	0.00146	0.00218	0.00652
AV35	-164.3869	54.8467	0.0039	0.00235	-0.00334	0.00146	0.00217	0.00636
AV36	-164.1268	54.7718	0.00655	-0.00304	-0.00166	0.00146	0.00217	0.00642
AV38	-163.7809	54.8315	-0.00125	-0.01445	0.00759	0.00148	0.00222	0.00668
AV39	-163.9985	54.8113	-0.00635	-0.02092	-0.00535	0.00148	0.00226	0.00666

Table S2.

Co-sesimic GNSS offsets processed by the Nevada Geodetic Laboratory (*84*)

(http://geodesy.unr.edu)

.

Station	Longitude (deg.)	Latitude (deg.)	E (m)	N (m)	U (m)	std E (m)	std N (m)	std U (m)
AB07	-160.4768	55.3493	0.058526	-0.024869	-0.007288	0.001259	0.001697	0.004413
AB13	-158.5038	56.3073	0.290613	-0.425627	-0.065849	0.002779	0.003615	0.002847
AB14	-159.0915	59.1082	0.007876	-0.038387	0.014963	0.001361	0.001576	0.004803
AC02	-154.1831	56.9506	-0.008018	-0.013314	0.00636	0.001547	0.001788	0.006569
AC08	-153.6447	58.9288	-0.002228	-0.009413	0.013224	0.001503	0.001078	0.004255
AC10	-164.8867	54.5226	-0.00319	-0.006407	0.008797	0.002196	0.001425	0.003795
AC12	-159.5896	54.831	-0.011729	-0.034531	-0.00856	0.001141	0.001541	0.002644
AC13	-155.6224	55.8219	0.291239	-0.261139	0.071691	0.003067	0.00251	0.002921
AC25	-162.3141	55.089	0.011903	-0.010412	0.001377	0.002477	0.001716	0.003045
AC26	-154.1503	58.2146	-0.010113	-0.015433	0.010177	0.000567	0.001677	0.004677
AC27	-154.1629	59.2525	-0.00536	-0.014954	0.011921	0.002473	0.002752	0.004858
AC28	-160.0492	55.0785	0.037188	-0.023973	-0.010094	0.001249	0.001566	0.003714
AC39	-152.3941	58.6097	-0.004795	-0.007359	0.014031	0.001075	0.001381	0.003964
AC40	-158.6186	56.9303	0.100849	-0.187647	0.003133	0.001619	0.001639	0.003325
AC41	-160.4073	55.9087	0.100521	-0.066053	-0.008635	0.000905	0.001598	0.006442
AV36	-164.1268	54.7718	0.001635	-0.008944	0.029688	0.001257	0.001032	0.010428

Table S3.

Cumulative co- and post-seismic displacement GNSS estimates.

Lon. (deg)	Lat. (deg)	Depth (km)	NP1 Strike	NP1 Dip	NP1 Rake	NP2 Strike	NP2 Dip	NP2 Rake	Moment (dyne-cm)
-157.32	55.4	30	238	10	88	60	80	90	2.95E+28
-156.87	55.27	20	219	24	55	77	71	105	2.04E+24
-156.79	55.25	34	237	22	78	70	69	95	2.05E+24
-154.19	55.4	15	248	9	107	51	82	88	6.66E+23
-157.45	55.17	25	238	11	86	63	79	91	3.96E+26
-156.23	54.91	21	230	17	73	68	74	95	3.21E+24
-156.31	54.95	12	228	11	83	55	79	91	1.04E+24
-156.4	56.01	39	72	52	-22	176	72	-140	4.71E+23
-158.66	54.86	45	248	27	95	63	63	88	5.59E+23
-158.52	54.79	33	240	22	82	69	68	93	4.15E+24

Table S4.

Post-seismic focal mechanisms from Harvard CMT project catalog (*95, 96*) in Global CMT convention. Columns are degrees longitude (deg.) , degrees latitude (deg.), depth (km), nodal plane 1 strike (deg.), dip (deg.), rake (deg.), nodal plane 2 strike (deg.), dip (deg.), rake (deg.), moment mantissa (dyne-cm), moment exponent.

REFERENCES

1. K. Schulz, *The Really Big One: An Earthquake Will Destroy a Sizable Portion of the Coastal Northwest. The Question Is When* (The New Yorker, 2015).

2. E. O. Lindsey, R. Mallick, J. A. Hubbard, K. E. Bradley, R. V. Almeida, J. D. P. Moore, R. Bürgmann, E. M. Hill, Slip rate deficit and earthquake potential on shallow megathrusts. *Nat. Geosci.* **14**, 321–326 (2021).

3. J. Hubbard, S. Barbot, E. M. Hill, P. Tapponnier, Coseismic slip on shallow décollement megathrusts: Implications for seismic and tsunami hazard. *Earth Sci. Rev.* **141**, 45–55 (2015).

4. R. von Huene, J. J. Miller, A. Krabbenhoeft, The Alaska convergent margin backstop splay fault zone, a potential large tsunami generator between the frontal prism and continental framework. *Geochem. Geophys. Geosyst.* **22**, e2019GC008901 (2021).

5. P. Segall, J. L. Davis, GPS applications for geodynamics and earthquake studies. *Annu. Rev. Earth Planet. Sci.* **25**, 301–336 (1997).

6. National Centers for Environmental Information, *National Geophysical Data Center/World Data Service: NCEI/WDS Global Historical Tsunami Database*, *2100 BC to Present*. (National Centers for Environmental Information, 2020)

7. Y.-J. Hsu, M. Simons, J.-P. Avouac, J. Galetzka, K. Sieh, M. Chlieh, D. Natawidjaja, L. Prawirodirdjo, Y. Bock, Frictional afterslip following the 2005 Nias-Simeulue earthquake, Sumatra. *Science* **312**, 1921–1926 (2006).

8. Y. Ito, T. Tsuji, Y. Osada, M. Kido, D. Inazu, Y. Hayashi, H. Tsushima, R. Hino, H. Fujimoto, Frontal wedge deformation near the source region of the 2011 Tohoku-Oki earthquake. *Geophys. Res. Lett.* **38**, L00G05 (2011).

9. C. Subarya, M. Chlieh, L. Prawirodirdjo, J. P. Avouac, Y. Bock, K. Sieh, A. J. Meltzner, D. H. Natawidjaja, R. McCaffrey, Plate-boundary deformation associated with the great Sumatra–Andaman earthquake. *Nature* **440**, 46–51 (2006).

10. J. F. Pacheco, L. R. Sykes, C. H. Scholz, Nature of seismic coupling along simple plate boundaries of the subduction type. *J. Geophys. Res. Solid Earth* **98**, 14133–14159 (1993).

11. M. Chlieh, J. P. Avouac, V. Hjorleifsdottir, T. R. A. Song, C. Ji, K. Sieh, A. Sladen, H. Hebert, L. Prawirodirdjo, Y. Bock, J. Galetzka, Coseismic slip and afterslip of the great M_w 9.15 Sumatra–Andaman earthquake of 2004. *Bull. Seismol. Soc. Am.* **97**, S152–S173 (2007).

12. L. L. Tsang *et al.*, Afterslip following the 2007 M_w 8.4 Bengkulu earthquake in Sumatra loaded the 2010 M_w 7.8 Mentawai tsunami earthquake rupture zone *J. Geophys. Res. Earth* **121**, 9034–9049 (2016).

13. T. Fujiwara, S. Kodaira, T. No, Y. Kaiho, N. Takahashi, Y. Kaneda, The 2011 Tohoku-Oki earthquake: Displacement reaching the trench axis. *Science* **334**, 1240 (2011).

14. Y. Ito, R. Hino, M. Kido, H. Fujimoto, Y. Osada, D. Inazu, Y. Ohta, T. Iinuma, M. Ohzono, S. Miura, M. Mishina, K. Suzuki, T. Tsuji, J. Ashi, Episodic slow slip events in the Japan subduction zone before the 2011 Tohoku-Oki earthquake. *Tectonophysics* **600**, 14–26 (2013).

15. K. Wang, T. Sun, L. Brown, R. Hino, F. Tomita, M. Kido, T. Iinuma, S. Kodaira, T. Fujiwara, Learning from crustal deformation associated with the M9 2011 Tohoku-oki earthquake. *Geosphere* **14**, 552–571 (2018).

16. J. E. Kozdon, E. M. Dunham, Rupture to the trench: Dynamic rupture simulations of the 11 March 2011 Tohoku earthquake. *Bull. Seismol. Soc. Am.* **103**, 1275–1289 (2013).

17. H. Noda, N. Lapusta, Stable creeping fault segments can become destructive as a result of dynamic weakening. *Nature* **493**, 518–521 (2013).

18. W. D. Barnhart, J. R. Murray, R. W. Briggs, F. Gomez, C. P. J. Miles, J. Svarc, S. Riquelme, B. J. Stressler, Coseismic slip and early afterslip of the 2015 Illapel, Chile, earthquake: Implications for frictional heterogeneity and coastal uplift. *J. Geophys. Res. Solid Earth* **121**, 6172–6191 (2016).

19. G. P. Hayes, G. L. Moore, D. E. Portner, M. Hearne, H. Flamme, M. Furtney, G. M. Smoczyk, Slab2, a comprehensive subduction zone geometry model. *Science* **362**, 58–61 (2018).

20. D. J. Wald, K. Jaiswal, K. Marano, D. Bausch, Earthquake impact scale. *Nat. Hazards Rev.* **12**, 125–139 (2011).

21. D. J. Wald, V. Quitoriano, T. H. Heaton, H. Kanamori, C. W. Scrivner, C. B. Worden, TriNet "ShakeMaps": Rapid generation of peak ground motion and intensity maps for earthquakes in southern California. *Earthq. Spectra* **15**, 537–555 (1999).

22. W. D. Barnhart, G. P. Hayes, D. J. Wald, Global earthquake response with imaging geodesy: Recent examples from the USGS NEIC. *Remote Sens. (Basel)* **11**, 1357 (2019).

23. C. Ji, D. J. Wald, D. V. Helmberger, Source description of the 1999 Hector Mine, California, earthquake, part I: Wavelet domain inversion theory and resolution analysis. *Bull. Seismol. Soc. Am.* **92**, 1192–1207 (2002).

24. D. E. Goldberg, P. Koch, D. Melgar, S. Riquelme, W. L. Yeck, Beyond the teleseism: Introducing regional seismic and geodetic data into routine USGS finite-fault modeling. *Seismol. Res. Lett.* **93**, 3308–3323 (2022).

25. C. J. Marone, C. H. Scholz, R. Bilham, On the mechanics of earthquake afterslip. *J. Geophys. Res.* **96**, 8441–8452 (1991).

26. J. R. Weiss, Q. Qiu, S. Barbot, T. J. Wright, J. H. Foster, A. Saunders, B. A. Brooks, M. Bevis, E. Kendrick, T. L. Ericksen, J. Avery, R. Smalley Jr, S. R. Cimbaro, L. E. Lenzano, J. Barón, J. C. Báez, A. Echalar, Illuminating subduction zone rheological properties in the wake of a giant earthquake. *Sci. Adv.* **5**, eaax6720 (2019).

27. B. A. Brooks, J. Murray, J. Svarc, E. Phillips, R. Turner, M. Murray, T. Ericksen, K. Wang, S. Minson, R. Burgmann, F. Pollitz, K. Hudnut, J. Nevitt, E. Roeloffs, J. Hernandez, B. Olson, Rapid geodetic observations of spatiotemporally varying postseismic deformation following

the Ridgecrest earthquake sequence: The U.S. Geological Survey response. *Seismol. Res. Lett.* **91**, 2108–2123 (2020).

28. J. E. Vidale, Y. G. Li, Damage to the shallow Landers fault from the nearby Hector Mine earthquake. *Nature* **421**, 524–526 (2003).

29. C. D. Chadwell, A. D. Sweeney, Acoustic ray-trace equations for seafloor geodesy. *Mar. Geod.* **33**, 164–186 (2010).

30. A. V. Newman, Hidden depths. *Nature* **474**, 441–443 (2011).

31. F. N. Spiess, C. D. Chadwell, J. A. Hildebrand, L. E. Young, G. H. Purcell Jr, H. Dragert, Precise GPS/Acoustic positioning of seafloor reference points for tectonic studies. *Phys. Earth Planet. In.* **108**, 101–112 (1998).

32. T. Sun, K. Wang, T. Fujiwara, S. Kodaira, J. He, Large fault slip peaking at trench in the 2011 Tohoku-oki earthquake. *Nat. Commun.* **8**, 14044 (2017).

33. M. Sato, T. Ishikawa, N. Ujihara, S. Yoshida, M. Fujita, M. Mochizuki, A. Asada, Displacement above the hypocenter of the 2011 Tohoku-Oki earthquake. *Science* **332**, 1395 (2011).

34. S.-i. Watanabe, T. Ishikawa, Y. Nakamura, Y. Yokota, Co- and postseismic slip behaviors extracted from decadal seafloor geodesy after the 2011 Tohoku-oki earthquake. *Earth Planets Space* **73**, 162 (2021).

35. J. L. Elliott, R. Grapenthin, R. M. Parameswaran, Z. Xiao, J. T. Freymueller, L. Fusso, Cascading rupture of a megathrust. *Sci. Adv.* **8**, eabm4131 (2022).

36. C. Liu, T. Lay, X. Xiong, The 29 July 2021 MW 8.2 Chignik, Alaska Peninsula earthquake rupture inferred from seismic and geodetic observations: Re-rupture of the Western 2/3 of the 1938 rupture zone. *Geophys. Res. Lett.*, e2021GL096004 (2022).

37. B. W. Crowell, D. Melgar, Slipping the Shumagin Gap: A kinematic coseismic and early afterslip model of the Mw 7.8 Simeonof Island, Alaska, earthquake. *Geophys. Res. Lett.* **47**, e2020GL090308 (2020).

38. M. W. Herman, K. P. Furlong, Triggering an unexpected earthquake in an uncoupled subduction zone. *Sci. Adv.* **7**, eabf759 (2021).

39. C. Drooff, J. T. Freymueller, New constraints on slip deficit on the aleutian megathrust and inflation at Mt. Veniaminof, Alaska from repeat GPS measurements. *Geophys. Res. Lett.* **48**, e2020GL091787 (2021).

40. I. E. Mulia, A. R. Gusman, M. Heidarzadeh, K. Satake, Sensitivity of Tsunami data to the up-dip extent of the July 2021 Mw 8.2 Alaska Earthquake. *Seismol. Res. Lett.* **93**, 1992–2003 (2022).

41. L. Ye, Y. Bai, D. Si, T. Lay, K. F. Cheung, H. Kanamori, Rupture model for the 29 July 2021 M_W 8.2 Chignik, Alaska earthquake constrained by seismic, geodetic, and tsunami observations. *J. Geophys. Res. Solid Earth* **127**, e2021JB023676 (2022).

42. J. T. Freymueller, E. N. Suleimani, D. J. Nicolsky, Constraints on the slip distribution of the 1938 M_w 8.3 Alaska Peninsula earthquake from Tsunami Modeling. *Geophys. Res. Lett.* **48**, e2021GL092812 (2021).

43. R. W. Briggs, S. E. Engelhart, A. R. Nelson, T. Dura, A. C. Kemp, P. J. Haeussler, D. R. Corbett, S. J. Angster, L. A. Bradley, Uplift and subsidence reveal a nonpersistent megathrust rupture boundary (Sitkinak Island, Alaska). *Geophys. Res. Lett.* **41**, 2289–2296 (2014).

44. S. Soloviev, Sanak-Kodiak tsunami of 1788. *Sci. Tsunami Haz.* **8**, 34–38 (1990).

45. J. F. Lander, *Tsunamis Affecting Alaska, 1737–1996* (U.S. Department of Commerce, National Oceanic and Atmospheric Administration, National Environmental Satellite, Data, and Information Service, National Geophysical Data Center, 1996).

46. R. C. Witter, R. W. Briggs, S. E. Engelhart, G. Gelfenbaum, R. D. Koehler, W. D. Barnhart, Little late Holocene strain accumulation and release on the Aleutian megathrust below the Shumagin Islands, Alaska. *Geophys. Res. Lett.* **41**, 2359–2367 (2014).

47. SAFRR Tsunami Modeling Working Group, "Modeling for the SAFRR Tsunami Scenario-Generation, propagation, inundation, and currents in ports and harbors; Chapter D, The SAFRR (Science Application for Risk Reduction) Tsunami Scenario" (U.S. Geological Survey Open-File Report 2013-1170, 2013); http://pubs.usgs.gov/of/2013/1170/d/.

48. K. Gagnon, C. D. Chadwell, E. Norabuena, Measuring the onset of locking in the Peru–Chile trench with GPS and acoustic measurements. *Nature* **434**, 205–208 (2005).

49. V. M. Cruz-Atienza, Y. Ito, V. Kostoglodov, V. Hjörleifsdóttir, A. Iglesias, J. Tago, M. Calò, J. Real, A. Husker, S. Ide, T. Nishimura, M. Shinohara, C. Mortera-Gutierrez, S. García, M. Kido, A seismogeodetic amphibious network in the Guerrero seismic gap, Mexico. *Seismol. Res. Lett.* **89**, 1435–1449 (2018).

50. T. Sun, K. Wang, Viscoelastic relaxation following subduction earthquakes and its effects on afterslip determination. *J. Geophys. Res. Solid Earth* **120**, 1329–1344 (2015).

51. T. Sun, K. Wang, T. Iinuma, R. Hino, J. He, H. Fujimoto, M. Kido, Y. Osada, S. Miura, Y. Ohta, Y. Hu, Prevalence of viscoelastic relaxation after the 2011 Tohoku-oki earthquake. *Nature* **514**, 84–87 (2014).

52. B. A. Brooks, L. N. Frazer, Importance reweighting reduces dependence on temperature in Gibbs samplers: An application to the coseismic geodetic inverse problem. *Geophys. J. Int.* **161**, 12–20 (2005).

53. S. E. Minson, M. Simons, J. L. Beck, Bayesian inversion for finite fault earthquake source models I—Theory and algorithm. *Geophys. J. Int.* **194**, 1701–1726 (2013).

54. W. Barnhart, R. Lohman, Automated fault model discretization for inversions for coseismic slip distributions. *J. Geophys. Res. Solid Earth* **115**, B10419 (2010).

55. U.S. Geological Survey National Earthquake Information Center, *M 8.2 - 99 km SE of Perryville, Alaska* (U.S. Geological Survey National Earthquake Information Center, 2021); https://earthquake.usgs.gov/earthquakes/eventpage/ak0219neiszm/executive.

56. K. S. Aslam, A. M. Thomas, D. Melgar, The effect of fore-arc deformation on shallow earthquake rupture behavior in the Cascadia subduction zone. *Geophys. Res. Lett.* **48**, e2021GL093941 (2021).

57. E. Suleimani, J. T. Freymueller, Near-field modeling of the 1964 Alaska tsunami: The role of splay faults and horizontal displacements. *J. Geophys. Res. Solid Earth* **125**, e2020JB019620 (2020).

58. A. Tsutsumi, O. Fabbri, A. M. Karpoff, K. Ujiie, A. Tsujimoto, Friction velocity dependence of clay-rich fault material along a megasplay fault in the Nankai subduction zone at intermediate to high velocities. *Geophys. Res. Lett.* **38**, L19301 (2011).

59. J. Wendt, D. D. Oglesby, E. L. Geist, Tsunamis and splay fault dynamics. *Geophys. Res. Lett.* **36**, L15303 (2009).

60. Y. Bai, K. F. Cheung, Y. Yamazaki, T. Lay, L. Ye, Tsunami surges around the Hawaiian Islands from the 1 April 2014 North Chile M_W 8.1 earthquake. *Geophys. Res. Lett.* **41**, 8512–8521 (2014).

61. M. Heidarzadeh, S. Murotani, K. Satake, T. Ishibe, A. R. Gusman, Source model of the 16 September 2015 Illapel, Chile, Mw 8.4 earthquake based on teleseismic and tsunami data. *Geophys. Res. Lett.* **43**, 643–650 (2016).

62. L. Li, T. Lay, K. F. Cheung, L. Ye, Joint modeling of teleseismic and tsunami wave observations to constrain the 16 September 2015 Illapel, Chile, M_w 8.3 earthquake rupture process. *Geophys. Res. Lett.* **43**, 4303–4312 (2016).

63. Y. Yamazaki, T. Lay, K. F. Cheung, H. Yue, H. Kanamori, Modeling near-field tsunami observations to improve finite-fault slip models for the 11 March 2011 Tohoku earthquake. *Geophys. Res. Lett.* **38**, L00G15 (2011).

64. L. Ye, H. Kanamori, T. Lay, Global variations of large megathrust earthquake rupture characteristics. *Sci. Adv.* **4**, eaao4915 (2018).

65. H. Yue, T. Lay, L. Rivera, Y. Bai, Y. Yamazaki, K. F. Cheung, E. M. Hill, K. Sieh, W. Kongko, A. Muhari, Rupture process of the 2010 M_w 7.8 Mentawai tsunami earthquake from joint inversion of near-field hr-GPS and teleseismic body wave recordings constrained by tsunami observations. *J. Geophys. Res. Solid Earth* **119**, 5574–5593 (2014).

66. S. Das, C. Henry, Spatial relation between main earthquake slip and its aftershock distribution. *Rev. Geophys.* **41**, 1013 (2003).

67. D. Drolet, M. G. Bostock, A. P. Plourde, C. G. Sammis, Aftershock distributions, moment tensors and stress evolution of the 2016 Iniskin and 2018 Anchorage M_w 7.1 Alaskan intraslab earthquakes. *Geophys. J. Int.* **231**, 199–214 (2022).

68. B. He, X. Wei, M. M. Wei, Y. Shen, M. Alvarez, A likely slow slip event detected by seafloor pressure data offshore southwest Alaska in 2018. *Abstracts AGU Fall Meeting* **2021**, T54A-06 (2021).

69. C. H. Scholz, Earthquakes and friction laws. *Nature* **391**, 37–42 (1998).

70. A. Bécel, D. J. Shillington, M. Delescluse, M. R. Nedimović, G. A. Abers, D. M. Saffer, S. C. Webb, K. M. Keranen, P.-H. Roche, J. Li, H. Kuehn, Tsunamigenic structures in a creeping section of the Alaska subduction zone. *Nat. Geosci.* **10**, 609–613 (2017).

71. J. Li, D. J. Shillington, A. Bécel, M. R. Nedimović, S. C. Webb, D. M. Saffer, K. M. Keranen, H. Kuehn, Downdip variations in seismic reflection character: Implications for fault structure and seismogenic behavior in the Alaska subduction zone. *J. Geophys. Res. Solid Earth* **120**, 7883–7904 (2015).

72. J. Li, D. J. Shillington, D. M. Saffer, A. Bécel, M. R. Nedimović, H. Kuehn, S. C. Webb, K. M. Keranen, G. A. Abers, Connections between subducted sediment, pore-fluid pressure, and earthquake behavior along the Alaska megathrust. *Geology* **46**, 299–302 (2018).

73. D. J. Shillington, A. Bécel, M. R. Nedimović, H. Kuehn, S. C. Webb, G. A. Abers, K. M. Keranen, J. Li, M. Delescluse, G. A. Mattei-Salicrup, Link between plate fabric, hydration and subduction zone seismicity in Alaska. *Nat. Geosci.* **8**, 961–964 (2015).

74. J. H. Dieterich, Earthquake nucleation on faults with rate-and state-dependent strength. *Tectonophysics* **211**, 115–134 (1992).

75. A. Ruina, Slip instability and state variable friction laws. *J. Geophys. Res.* **88**, 10359–10370 (1983).

76. P. M. Fulton, E. E. Brodsky, Y. Kano, J. Mori, F. Chester, T. Ishikawa, R. N. Harris, W. Lin, N. Eguchi, S. Toczko; Expedition 343, 343T, and KR13-08 Scientists, Low coseismic friction on the Tohoku-Oki fault determined from temperature measurements. *Science* **342**, 1214–1217 (2013).

77. E. L. Evans, S. E. Minson, C. D. Chadwell, Imaging the next Cascadia earthquake: Optimal design for a seafloor GNSS-A network. *Geophys. J. Int.* **228**, 944–957 (2022).

78. C. Liu, T. Lay, X. Xiong, Y. Wen, Rupture of the 2020 M_w 7.8 earthquake in the Shumagin gap inferred from seismic and geodetic observations. *Geophys. Res. Lett.* **47**, e2020GL090806 (2020).

79. J. Lin, R. S. Stein, Stress triggering in thrust and subduction earthquakes and stress interaction between the southern San Andreas and nearby thrust and strike-slip faults. *J. Geophys. Res. Solid Earth* **109**, B02303 (2004).

80. H. F. Ryan, R. E. von Huene, R. E. Wells, D. W. Scholl, S. Kirby, A. E. Draut, “History of earthquakes and tsunamis along the eastern Aleutian-Alaska megathrust, with implications for tsunami hazards in the California Continental Borderland” (U.S. Geological Survey Professional Paper 1795-A, 2012).

81. T. Dura, A. J. Garner, R. Weiss, R. E. Kopp, S. E. Engelhart, R. C. Witter, R. W. Briggs, C. S. Mueller, A. R. Nelson, B. P. Horton, Changing impacts of Alaska-Aleutian subduction zone tsunamis in California under future sea-level rise. *Nat. Commun.* **12**, 7119 (2021).

82. R. Bürgmann, D. Chadwell, Seafloor geodesy. *Annu. Rev. Earth Planet. Sci.* **42**, 509–534 (2014).

83. T. Nishimura, M. Sato, T. Sagiya, Global Positioning System (GPS) and GPS-Acoustic observations: Insight into slip along the subduction zones around Japan. *Annu. Rev. Earth Planet. Sci.* **42**, 653–674 (2014).

84. G. Blewitt, W. C. Hammond, C. Kreemer, Harnessing the GPS data explosion for interdisciplinary science. *Eos* **99**, 485 (2018).

85. G. P. Hayes, Rapid source characterization of the 2011 M_w 9.0 off the Pacific coast of Tohoku earthquake. *Earth Planets Space* **63**, 529–534 (2011).

86. D. E. Goldberg, W. D. Barnhart, B. W. Crowell, *Regional and Teleseismic Observations for Finite-Fault Product* (U.S. Geological Survey Data Release Products, 2022); https://doi.org/10.5066/P9ZO5FRS.

87. M. Bevis, S. J. Martel, Oblique plate convergence and interseismic strain accumulation. *Geochem. Geophys. Geosyst.* **2**, 1033 (2001).

88. M. Comninou, J. Dundurs, The angular dislocation in a half space. *J. Elast.* **5**, 203–216 (1975).

89. M. Jeyakumaran, J. W. Rudnicki, L. M. Keer, Modeling slip zones with triangular dislocation elements. *Bull. Seismol. Soc. Am.* **82**, 2153–2169 (1992).

90. L. Langer, H. N. Gharti, J. Tromp, Impact of topography and three-dimensional heterogeneity on coseismic deformation. *Geophys. J. Int.* **217**, 866–878 (2019).

91. Z. Duputel, P. S. Agram, M. Simons, S. E. Minson, J. L. Beck, Accounting for prediction uncertainty when inferring subsurface fault slip. *Geophys. J. Int.* **197**, 464–482 (2014).

92. T. C. Hanks, H. Kanamori, A moment magnitude scale. *J. Geophys. Res. Solid Earth* **84**, 2348–2350 (1979).

93. U.S. Geological Survey National Earthquake Information Center, *M 7.8 - 99 km SSE of Perryville, Alaska* (U.S. Geological Survey National Earthquake Information Center, 2020); https://earthquake.usgs.gov/earthquakes/eventpage/at00qdux98/executive.

94. U.S. Geological Survey National Earthquake Information Center, *M 7.6 - 99 km SE of Sand Point, Alaska* (U.S. Geological Survey National Earthquake Information Center, 2020); https://earthquake.usgs.gov/earthquakes/eventpage/us6000c9hg/executive.

95. A. M. Dziewonski, T. A. Chou, J. H. Woodhouse, Determination of earthquake source parameters from waveform data for studies of global and regional seismicity. *J. Geophys. Res. Solid Earth* **86**, 2825–2852 (1981).

96. G. Ekström, M. Nettles, A. M. Dziewoński, The global CMT project 2004–2010: Centroid-moment tensors for 13,017 earthquakes. *Phys. Earth Planet. In.* **200–201**, 1–9 (2012).

97. A. R. Nelson, R. W. Briggs, T. Dura, S. E. Engelhart, G. Gelfenbaum, L.-A. Bradley, S. L. Forman, C. H. Vane, K. A. Kelley, Tsunami recurrence in the eastern Alaska-Aleutian arc: A holocene stratigraphic record from Chirikof Island, Alaska. *Geosphere* **11**, 1172–1203 (2015).
